# Green Synthesis of Silver Nanoparticles Using *Annona muricata* Extract as an Inducer of Apoptosis in Cancer Cells and Inhibitor for NLRP3 Inflammasome via Enhanced Autophagy

**DOI:** 10.3390/nano11020384

**Published:** 2021-02-03

**Authors:** Majid S. Jabir, Yasmin M. Saleh, Ghassan M. Sulaiman, Nahi Y. Yaseen, Usama I. Sahib, Yaser Hassan Dewir, Mona S. Alwahibi, Dina A. Soliman

**Affiliations:** 1 Department of Applied Sciences, University of Technology, Baghdad 10066, Iraq; usamaimad3@gmail.com; 2 College of Education, Mustansiriyah University, Baghdad 10052, Iraq; yasmin_angel89@yahoo.com; 3 Iraqi Center for Cancer and Medical Genetics Research, Mustansiriyah University, Baghdad 10052, Iraq; nahiyaseen@iccmgr.org; 4 Plant Production Department, P.O. Box 2460, College of Food and Agriculture Sciences, King Saud University, Riyadh 11451, Saudi Arabia; ydewir@ksu.edu.sa; 5 Faculty of Agriculture, Kafrelsheikh University, Kafr El-Sheikh 33516, Egypt; 6 Department of Botany and Microbiology, P.O. Box 22452, College of Science, King Saud University, Riyadh 11495, Saudi Arabia; malwhibi@ksu.edu.sa (M.S.A.); dsoliman@ksu.edu.sa (D.A.S.)

**Keywords:** *Annona muricata*, silver nanoparticle, anticancer, apoptosis, autophagy, IL-1- β, NLRP3

## Abstract

*Annona muricata* is one of the most important traditional medicinal plants which contains numerous chemicals that exhibit various pharmacological properties. In this study, silver nanoparticles were prepared using *A. muricata* peel extract as a reducing agent and the effect was enhanced through *A. muricata* like pharmaceutical activity. AgNPs formation was confirmed by color changes, UV-visible spectroscopy, SEM, DLS, and XRD. The anti-proliferative activity of AgNPs against THP-1, AMJ-13, and HBL cell lines was studied. Apoptotic markers were tested using AO/EtBr staining assay, cell cycle phases using flowcytometry, and the expression of P53. Autophagy takes an essential part in controlling inflammasome activation by primary bone marrow-derived macrophages (BMDMs). We report novel functions for AgNPs-affected autophagy, represented by the control of the release of IL-1β, caspase-1, adaptor protein apoptosis-associated speck-like protein containing a CARD (ASC), and NLRP3 in BMDMs following treatment with LPS+ATP. The current study revealed that the AgNPs inhibited THP-1 and AMJ-13 cell proliferation. Meanwhile, the AgNPs significantly increased autophagy and reduced IL-1b and NLRP3 levels in both in vivo and in vitro models. The secretion of IL-1β was reduced whereas the degradation of NLRP3 inflammasome was enhanced. These findings propose that AgNPs apply an anti-proliferative activity against THP-1 and AMJ-13 cells through the stimulation of apoptosis via mitochondrial damage and induction of p53 protein pathway. In addition, AgNP-induced autophagy reduced the levels of IL-1β and NLRP3 inflammasome activation. This indicated that the AgNPs augment autophagy controlled by the IL-1β pathway via two different novel mechanisms. The first one is regulating activation of the IL-1 β, caspase-1, and ASC, while the second is NLRP3 targeting for lysosomal degradation. Overall, this study suggests that AgNPs could be a potent therapy for various types of cancer and an alternative treatment for preventing inflammation via enhancing autophagy.

## 1. Introduction

*Annona muricata* (graviola) is indigenous to the Amazon as well as most of tropical regions of South and North America, This species is extensively used in traditional medicine against various conditions, including cancer and parasitic infections [1]. The excess in the production of free radicals is among the main indicators of malignant cell development. A number of investigations demonstrated that antioxidants produced by plants can act in scavenging free radicals and regulating the impacts associated with oxidative stress [2]. The effectiveness of different plant-extracted materials toward proliferating cells is well established. Their cytotoxic impacts are indicated by the damage they cause to DNA or the blockage of mitotic spindle synthesis in the course of the process of cell division [3]. However, most cytotoxic chemopreventive drugs exhibit toxicity during therapy. Thus, there is a necessity to manufacture new materials that have both strength and selectivity, while their side effects against normal cells must be at the minimum [4].

Cancer remains a life-threatening disease with high mortality rates worldwide. Data recently released by Iraq’s National Cancer Registry revealed more than 31,500 cancer and tumor-associated cases in 2017–2018. Cancer is locally regarded as being among the main contributors to mortality, accounting for approximately 11% of the total rate [5]. Genetic factors are associated with 5–10% of cancers, while other factors also make contributions, such as insufficient diet, certain infections, physical inactivity, obesity, smoking, as well as air and water pollution, which can exert direct or indirect impacts on the activity of key cancer-related genes [6]. 

Compounds extracted from plants have emerged as a promising approach to greenly-synthesize metallic nanoparticles (NPs) [7]. Hence, it is still a challenging task to greenly-synthesize NPs while maintaining certain shape, size, and distribution conditions [8]. The use of classical chemical medications have been associated with an increasing number of various limitations. These drugs are low-specific, irregularly distributed in organs and tissues, and readily cleared and biodegraded [9]. Such an area of research involves the application of nanotechnology to human health with the provision of novel methodologies for the treatment of various human illnesses, including cancer [10]. Most of the nanomaterials exert distinctive characteristics that ensure usefulness in various applications of biotechnology, leading to their exploitation in the innovation of highly-effective tools used both for diagnosis and treatment [11,12]. Nanomaterials can also be utilized as drug delivery systems, leading to the reduction of the undesired effects usually associated with the use of conventional drugs [13,14]. In the near future, an increasing number of these materials is expected to have the approval by the Food and Drug Administration (FDA) [15,16]. 

There are several studies demonstrated that the AgNPs had anticancer activity. A study by Saratale et al. [17] the prepared AgNPs using *Taraxacum officinale* showed their high cytotoxic effect against human liver cancer cells (HepG2). While, The AgNPs prepared using *Commelina nudiflora* aqueous extract showed a high cytotoxic activity against HCT-116 colon cancer cells [18]. AgNPs synthesized by other plants exhibited the anti-proliferative activity against human colorectal adenocarcinoma, the human kidney, human chronic myelogenous, leukemia, bone marrow, and human cervix [19]. The developed AgNPs using *Nostoc linckia* had been showed anticancer activity against breast cancer cell MCF-7, The (IC_50_) was 27.79 ± 2.3 μg/ mL [20]. Additionally, the chemically synthesized AgNPs composites possessed promising anticancer activity against the A549, HeLa cells, MCF7 cells, MDAMB231, and SKBR3 cells [21]. 

Apoptosis is a mechanism with a high level of regulation where a sequence of molecular events is activated and result in cell death. Two main pathways are responsible for the commence of apoptosis, which are extrinsic pathway, mediated by the death receptor, and the intrinsic pathway, mediated by events that take place in the mitochondria. The latter of these is predominantly controlled via the Bcl-2 family of proteins, characterized by its two functional groups of pro-apoptotic (e.g., Bax and Bid) and anti-apoptotic (e.g., Bcl-2 and Bcl-xL) molecules [22]. Remarkably, different nanomaterials have been described to be capable of exerting cytotoxic impacts via the modulation of the autophagy process [23,24]. For instance, AgNPs were demonstrated to cause impairment of monocyte–macrophage differentiation via the blocking of autophagy, that is regulated by lysosomal dysfunction. Autophagy is a process of self-degradative in response to stresses such as starvation by which damaged macromolecule, micromolecules, and organelles are targeted by autophagic vesicles to lysosomes and then removed. Indeed, impairment of lysosomes was described in THP-1 cells treated with AgNPs where blocking of autophagic flux [25] was also being involved. The same study demonstrated the existence of a simultaneously AgNPs-stimulated crosstalk that involves the processes of monocyte differentiation, autophagy, and lysosomal dysfunction. Nevertheless, recent studies have demonstrated that autophagy plays a significant role in anticancer therapy, and the enhancement of autophagy and apoptosis leads to inhibit tumorigenesis [26,27]. So, the stimulation of autophagy is currently considered a novel therapeutic method. Green synthesis enables the creation of nanoparticles via a reaction that involves naturally-occurring materials which act as reducing agents and metal salts. When plant extracts are employed, they act as reducing and stabilizing agents [28]. The current study was established with the goal of preparing silver nanoparticles using *Annona muricata* as a reducing agent and investigating its activity as a new therapeutic approach against cancer. In particular, the study investigated the ability of the synthesized nanoparticles to reduce NLRP3 inflammasome activity through the induction of autophagy. 

## 2. Materials and Methods 

### 2.1. Cells and Reagents

The THP-1 cells are designates a spontaneously immortalized monocyte-like cell line, derived from the peripheral blood of a childhood case of acute monocytic leukemia, AMJ-13 cells are Iraqi patient breast cancer cell line it is a receptor-negative, hormone-nonresponsive, and HBL cells are breast epithelial cell line. All cells were kindly provided by the Iraqi Centre for Cancer and Medical Genetic Research (ICCMGR), Al-Mustansiriyah University, Baghdad, Iraq. RPMI-1640, trypsin-EDTA, dimethyl sulfoxide (DMSO), fetal bovine serum, ovalbumin, 3-(4,5-dimethylthiazal-z-yl)-2,5-diphenylterazolium (MTT), lipopolysaccharides (LPS) from Escherichia coli O111:B4 (Cat. No. L2630, Sigma- Aldrich, MO, USA), adenosine triphosphate (ATP), and Triton X-100 were from Sigma (MO, USA). 3,3′,5,5′-Tetramethylbenzidine (TMB) microwell peroxidase substrate solution was obtained from Kirkegaard & Perry Laboratories, Inc. (KPL) (Gaithersburg, MD, USA). Stop solution was obtained from eBioscience (Wembley, UK). The remaining chemicals and reagents were of analytical grade.

### 2.2. Extraction of Plants

*Annona muricata* peels were collected from local market at Diyala province, Iraq, dried and ground into powder. Subsequently, 50 g of the powder was soaked in a flask containing 500 mL of deionized distilled water. Next, extraction was performed via incubation in an orbital shaker at room temperature (RT) with agitating the flasks (100 rpm, 72 h). Following the extraction, filtration of the aqueous solution of peel extract was achieved using Whatman filter paper No. 1. Then, crude extract was obtained via the concentration of extract with the use of reduced pressure. Subsequently, the extract was stored at 4 °C until used [29].

### 2.3. Preparation of Silver Nanoparticles 

The synthesis of AgNPs was performed using the green synthesis method that was previously prepared by Gavamukulya et al. [30] after applying slight modifications. Aliquots of 9 mL of 2 mM silver nitrate were added to 1 mL *A. muricata* peel extract and following incubation for 8 h at RT, the bioreduction of AgNO_3_ was achieved.

### 2.4. Characterization of Silver Nanoparticles

The synthesis of AgNPs was confirmed utilizing UV-visible spectroscopic analysis (Hitachi U-2910 Spectrophotometer, Hitachi Ltd., Tokyo, Japan) where the NPs were continuously scanned at 280–760 nm. Morphological characterization of the NPs was performed via scanning electron microscopy (SEM, AA-7000; Shimadzu, Tokyo, Japan). The crystalline nature of the nanoparticles was confirmed using an XRD-6000 X-ray diffractometer (Shimadzu, Tokyo, Japan) by applying a voltage of 40 kV and current of 30 mA, with CuKα radiation in 2θ configurations [31,32,33]. We confirmed the formation of AgNPs using the DLS assay, depending on light supply and the type of the detector; distilled water (1 mL) was pipetted into the cells, then 50 μL of the stock dispersions was applied. Thereafter, the DLS method was employed for the identification of the distribution pattern of the length of the NPs.

### 2.5. Lactate Dehydrogenase Release Assay

Lactate dehydrogenase (LDH) releasing activity assay was tested following the guidelines specified by the manufacturer. The cells were treated with AgNPs for 24, 48, and 72 h. Then, the supernatant of the treated cells was transferred into a 96-well plate to evaluate LDH release activity by measuring optical density at 490 nm.

### 2.6. Cell Proliferation Assay

The proliferation of the Ag-NPs-treated THP-1, AMJ-13, and HBL cells was investigated. Labeling of the cells was performed using the cell proliferation kit for flow cytometry (CellTrace™ Red-APC) prior to treatment with the AgNPs, in accordance with the kit protocol (Gibco/Thermo Fisher Scientific, Waltham, MA, USA) [34]. 

### 2.7. MTT Assay 

The cytotoxicity test was performed based on a previously reported protocol [35]. Seeding of the cells was conducted at a concentration of 1 × 10^5^ cells mL^−1^ in wells of microtiter plates supplied with RPMI. The cells were allowed to adhere overnight, following by the application of different concentrations of AgNPs in triplicate and incubation for various periods (24, 48, and 72 h). Thereafter, the MTT solution was applied to the cells which were incubated for the set time. After the aspiration of the medium and the addition of DMSO medium, absorbance was determined (492 nm) on a microplate reader. Regression analysis was used to extract the concentration required to achieve 50% inhibition of cell growth (IC_50_) [36,37]. 

### 2.8. Clonogenicity Assay

THP-1 and AMJ-13 cells were cultured in 24-well plates (10^5^ cell/mL) for 24 h, followed by treatment with AgNPs. Cells were removed from the medium after reaching monolayer confluence and rinsed in PBS. The cells were then fixed, followed by staining with crystal violet (Sigma-Aldrich, MO, USA), washing to remove excess dye, and photography [38]. 

### 2.9. Acridine Orange–Ethidium Bromide Staining

AO/EtBr staining (Sigma-Aldrich, USA) was performed to measure the induction of apoptosis in AgNP-treated cells. Briefly, after the cells were seeded in 12-well plates for 24 h, treatment with AgNPs was applied. The cells were washed twice with PBS. A mixture of equal volumes of the cells and the dual fluorescent dyes (10 µL) was prepared, followed by examination under fluorescence microscopy [39]. 

### 2.10. Flow Cytometry Assay for Apoptotic Cells

Cell cycle phase determination kit was employed for the detection of cells in the sub-G_1_. Briefly, THP-1 and AMJ-13 cells were plated at a concentration of 1 × 10^6^ cell mL^−1^. After treatment with AgNPs for 24 h., the cells were centrifuged, washed with PBS, fixed, and resuspended in staining buffer that contains propidium iodide (PI) and RNase A. The FL2 channel of the flow cytometer was utilized to detect the sub-G_1_ peak. 

### 2.11. Mitochondrial Membrane Potential Assay

Rhodamine (Rh123) is a fluorochrome that is retrieved by lively mitochondria with no cytotoxic impacts. This stain was employed to study the membrane potential of mitochondria prior to and following the application of AgNPs to the cells. In brief, THP-1 and AMJ-13 were seeded in 96-well plates at a concentration of 10^4^ for 24 h. After the cells being treated with AgNPs, they were dyed with Rh123 at a concentration of 10 M (1 h, 37 °C). Trypsin-EDTA (0.25%) was applied to detach the cells, followed by centrifugation at 500 rpm for 2 min. Cells were resuspended in FACS buffer and detected using flow cytometry assay, and histograms were created [40]. 

### 2.12. Real-Time PCR 

THP-1 and AMJ-13 cells were tested to determine the expression level of p53 after treatment with AgNPs. The primer sets were established according to the standard sequences published in the NCBI database. The primers were utilized for the quantitative RT-PCR assay, with the following sequences: P53 forward: 5′-CCGTCCCAAGCAATGGATG-3′, reverse: 5′-GAAGATGACAGGGGCCAGGAG-3′. Following purification of total RNA (RNeasy Mini Kit, Cat. No. 74104; Qiagen, UK) and treatment with DNase, treated with Superscript II reverse transcriptase was applied to synthesize cDNA (Cat. No. 18064-071; Invitrogen, USA). For quantitative reverse-transcription polymerase chain reaction (qRT-PCR), each reaction involved a mixture of of cDNA product (1 μL), SYBR green dye (7.5 μL), ROX (0.3 μL), and specified primers (0.3 μL); the final volume was completed to 15 μL via the addition of 5.6 μL of distilled water. Then, fast SYBR Green master mix was added (Cat. No. 4385612; Applied Biosystems, London, UK) in addition to the 7900HT fast system (Applied Biosystems). After gene expression levels were normalized utilizing TBP protein, housekeeping genes were used as reference genes for the normalization of expression values of target genes. Mean relative expression values were determined according to previously described approaches [41]. 

### 2.13. Anti-Inflammatory Activity of Silver Nanoparticles: An In Vitro Model 

#### 2.13.1. Bone Marrow-Derived Macrophages

Male C57/BL6 mice of 8–10 weeks of age were used to isolate primary bone marrow-derived macrophages (BMDMs). After isolation, the BMDM cells were exposed to LPSs and ATP treatment, with and without AgNPs, to study their ability to reduce IL-1β secretion and NLRP3 inflammasome activation. 

#### 2.13.2. Anti-Inflammatory Activity of Silver Nanoparticles: An In Vivo Model 

Male mice (8–10 weeks old) were randomly divided into three groups, each containing six mice. All animals were kept under standard environmental conditions of ~23–27 °C, humidity of ~55–60%, and~12 h light/dark cycle. The animals were kept in polypropylene cages with wood dust and ad libitum food and water. Approval of experimental protocols was obtained from the Animal Ethical Committee at the Division of Biotechnology, Department of Applied Sciences, University of Technology, Baghdad, Iraq. The first group was intraperitoneally injected (IP) with 250 µL of normal saline, the second group was injected IP with 50 µg of LPS for 8 h, and the third group was pretreated with AgNPs at a concentration of 500 µg kg^−1^ and then injected IP with 50 µg of LPS for 8 h. Animals were sacrificed, after which their blood was collected by cardiac puncture for the analysis of IL-1β. 

#### 2.13.3. Enzyme-Linked Immunosorbent Assay

The cytokine concentration of mouse IL-1β (Cat. No. ab1977742; Abcam, Cambridge, MA, USA) was estimated using an enzyme-linked immunosorbent assay (ELISA) kit, according to the manufacturer’s guidance. The absorbance was read using an ELISA plate reader at 570 nm [42]. 

### 2.14. Immunofluorescence Microscopy Assay

Seeding of THP-1 and AMJ-13 cells was achieved onto plastic Lab-tek two-well slides. Then, the cells were exposed to subsequent treatments with (500 µgmL^−1^ for 24 h) and ATP (5 mM/mL for 30 min), with or without treatment with the prepared AgNPs. The cells were then subjected to standard steps of washing (PBS for three times), fixation (4% PFA, 30 min, RT), permeabilization (0.5% Triton-X, 30 min, RT), and blocking (10% normal goat serum, 60 min). Next, the cells were treated with 1 µgmL^−1^ of each of the primary rabbit polyclonal anti-LC3, rabbit polyclonal anti-NLRP3, anti-Caspase-1, and anti-ASC antibodies for 24 h at 4 °C and washed two times with PBS. Then, the cells were treated with 1 µgmL^−1^ of the secondary antibodies Alexa Fluor 488-conjugated goat anti-rabbit IgG or Alexa Fluor 568-conjugated goat anti-mouse IgG for 2 h at RT. The cells were washed thrice using PBS and mounted in Vectashield with DAPI. Finally, they were examined under a confocal microscope. 

### 2.15. Flow Cytometry Assay for LC3, NLRP3, IL-1β, and Caspase-1 

Non-specific Fc-mediated binding between the antibodies and Fc receptors was blocked with 1 µgmL^−1^ rat anti-mouse CD16/CD32 antibody for 30 min at 4 °C. LC3, NLRP3, IL-1β, and Caspase-1 were measured using flow cytometry assay [43] (FACS Calibur flow cytometer, BD). 

### 2.16. ROS Detection Assay 

Intracellular production of ROS was estimated using flow cytometry assay according to the manufacturer’s protocol. Briefly, the cells were seeded (1 × 10^5^ cells/well), followed by treatment LPS + ATP, with and without AgNPs treatment. After harvesting, incubation with Cell ROX Orange Reagent was performed for 30 min. Additional washing and harvesting steps were conducted prior to the analysis of intracellular fluorescence (FACS Calibur flow cytometer). 

### 2.17. Western Blot Assay 

The immunoblot test was conducted based on the supplier’s protocol. The cells were subjected to treatment with LPS + ATP for 6 h, with and without treatment with AgNPs. Cell lysis was performed for 20 min with protease inhibition buffer, followed by centrifugation (15,000 rpm, 10 min) for cell lysate collection. Measurement of protein concentration was performed by utilizing Micro BCA™ Protein Assay Reagent Kit. Lysate dilution was conducted with LDS buffer along with reducing agent. Samples were mounted on to NU-PAGE Bis-Tris gels, which were run at 120 V and 150 mA, and then placed onto PVDF (Hybond-P polyvinylidenedifluoride) membrane by means of Hoefer TE 22 tank transfer unit along with NuPAGE transfer buffer provided with 20% methanol (90 V, 125 mA, 2 h, RT). Dried skimmed milk (5%) was employed for 60 min for blocking the PVDF membrane, after which the samples were incubated (4 °C) in shaking incubator. Primary polyclonal antibodies (Abs) for LC3 and NLRP3 proteins were applied (1 µg mL^−1^, 2 h, room temperature) and the unbound ones were removed via washing thrice with PBS. Next, incubation of the membrane was performed with 1 µg mL^−1^ anti-rabbit IgG HRP-linked Ab (60 min, RT). Another washing step of unbound secondary Abs was conducted, while the bound ones were measured by an enhanced chemi-luminescence kit and exposed to an X-ray film for visualization. 

### 2.18. Statistical Analysis 

Unpaired *t*-test (GraphPad Prism 5) was applied to statistically process the data, with the results shown as the mean ± standard error of the mean of three replicates per assay [43].

## 3. Results and Discussion

### 3.1. Characterization of the Prepared AgNPs 

**Figure 1:**
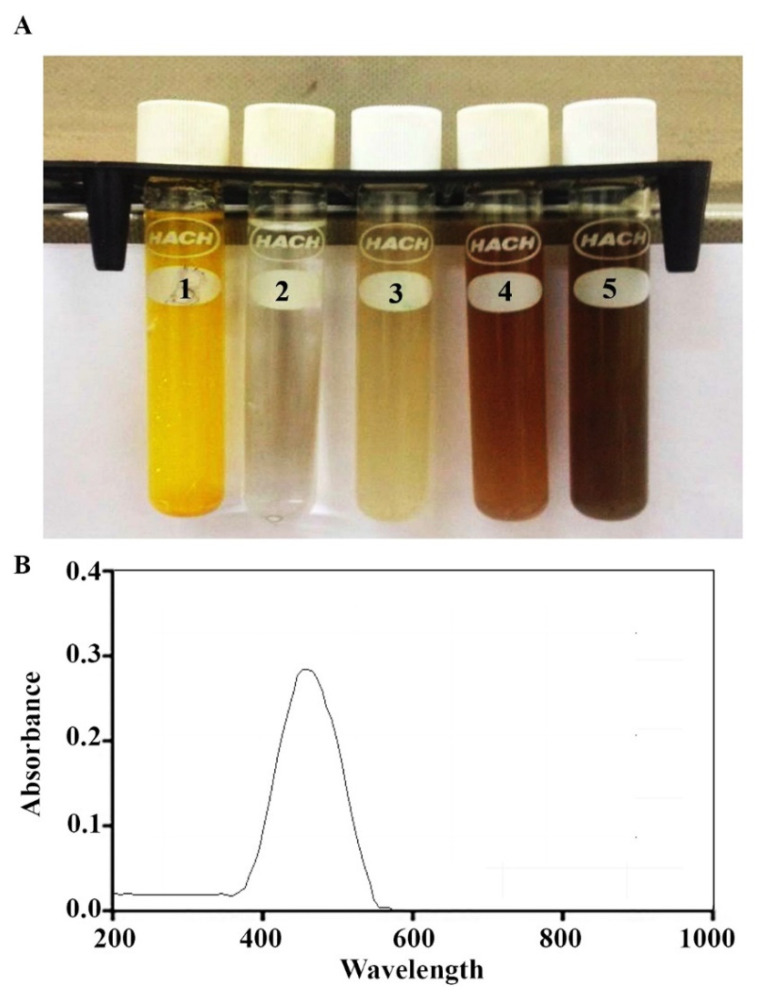
Formation of AgNPs. (**A**) Color changes: 1, *Annona muricata* peel extract; 2, silver nitrate solution; 3, silver nanoparticles at a concentration of 1 mM of AgNO_3_; 4, silver nanoparticles at a concentration of 2.5 mM of AgNO_3_; and 5, silver nanoparticles at a concentration of 5 mM of AgNO_3_. (**B**) UV-spectroscopy of AgNPs.

**Figure 2:**
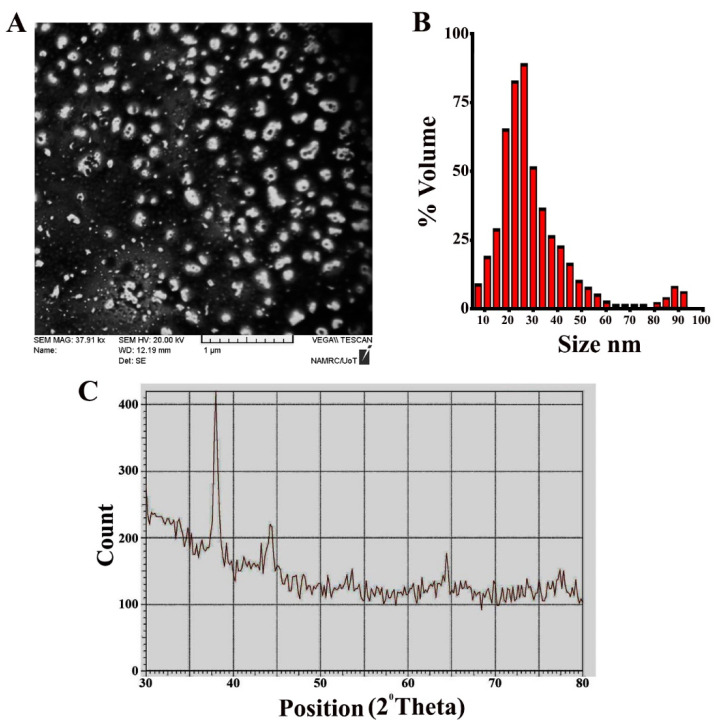
Morphological and structural characteristics of AgNPs. (**A**) SEM image. (**B**) DLS. (**C**) XRD pattern.

The *Annona muricata* peel extract was deep yellow. Its color changed to dark brown after silver nitrate was added (1 mM/mL for 10 h). The reduction of Ag^+^ into NPs during exposure to *Annona muricata* peel extract is shown in Figure 1A. UV spectroscopy was also used to analyze the synthesized AgNPs. As seen in Figure 1B, the peak of the synthesized AgNPs was noticed at approximately 430 nm. Meanwhile, the results of SEM imaging demonstrated the synthesis of nanoparticles with diameters of 11–23 nm (Figure 2A). DLS assay was conducted to determine the Brownian movement of the dispersed NPs that are spherical in shape. The assay was also performed to draw a relationship between this behavior and the hydrodynamic length of the NPs within the dispersing solution. Mathematic computation of the dynamic variations of the scattered light intensity can be conducted in order to find the relation to the hydrodynamic length of the NPs. As shown in Figure 2B, the AgNPs showed diameter values which approximately ranged 16–31 nm. The XRD pattern obviously demonstrated that the the AgNPs synthesized through the reduction of Ag ions by *Annona muricata* peel extract are crystalline in nature (Figure 2C).

### 3.2. AgNPs Increase Lactate Dehydrogenase Release 

**Figure 3:**
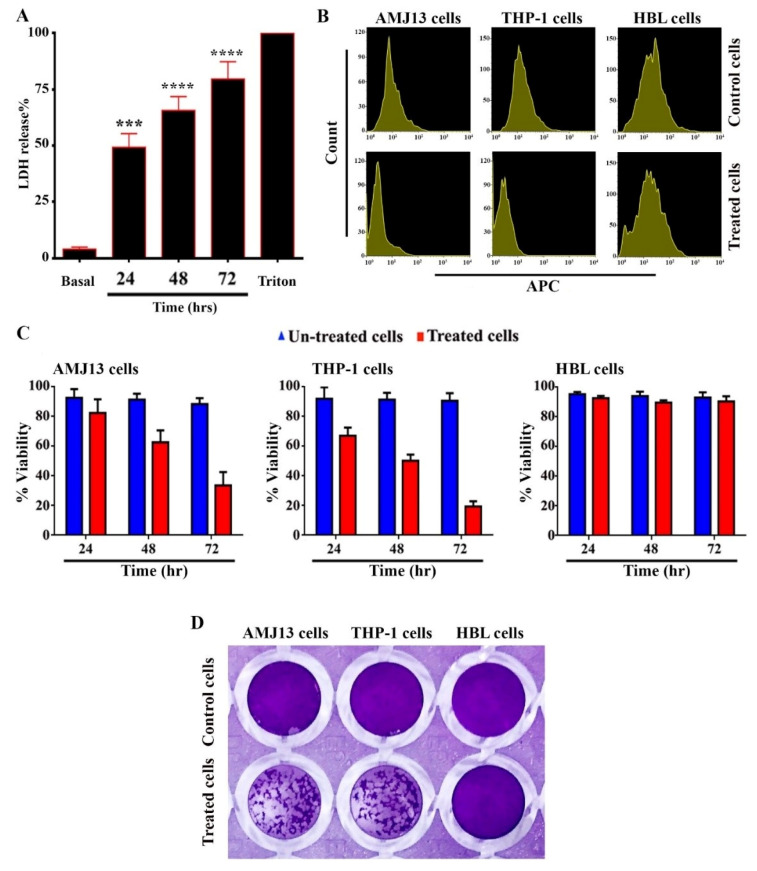
Anti-proliferative activity of AgNPs against AMJ-13 and THP-1 cells. (**A**) AgNPs increase lactate dehydrogenase (LDH) release. The cells were exposed to AgNPs (25 µg cells mL^−1^ for 24, 48, and 72 h). 1% Triton-x was utilized as a positive control. The LDH release activity was measured at 490 nm. Results are represented as mean ± SD *** *p* < 0.001, **** *p* < 0.0001. (**B**) Representative proliferation assay by CellTrace™, APC meaning Allophycocyanin. (**C**) Cytotoxic activity of AgNPs against cancer cells in different times. THP-1, AMJ-13, and HBL cells were seeded at a concentration of 1 × 10^5^ cells mL^−^^1^. AgNPs were added in triplicate and incubated for 24, 48, and 72 h. MTT stain was applied to the cells. Following incubation for 3 h at 37 °C. DMSO added to samples. The absorbance was determined at a wavelength of 492 nm. (**D**) Colony-forming unit assay of AMJ-13, THP-1, and HBL cells. Cells were treated with AgNPs at IC_50_ (17.34 µgmL^−1^) for 24 h, then stained with crystal violet stain for 20 min.

The conversion of lactate to pyruvate in cells depends on the enzyme lactate dehydrogenase, which is a significant step for energy production in cells. When this enzyme is released into culture medium containing cells treated with certain substances, this indicates a loss of cell membrane integrity. LDH test was employed to measure the cytotoxic impact of AgNPs on treated cells. Lactate dehydrogenase is released into the cytoplasm after cell damage, which causes tetrazolium salt to convert into formazan. Formazan production measured at 490 nm is reflective of the number and proportion of damaged, injured, or dying cells. The obtained data could be revealed the capability of the AgNPs to penetrate treated cells, stimulate vesicle formation, and enter vesicles. Figure 3A showed the ability of AgNPs in increasing of LDH release. The results is time dependent manner. Small silver nanoparticles can easily enter cells and other biological systems and cause substantial cellular damage, leading to the release of LDH. However, nanoparticles of 100–200 nm can also be taken up by cells and provoke toxic reactions, including DNA damage and genetic alteration. The toxicity of nanoparticles could be related to their ability to disrupt the antioxidant system and induce oxidative stress [44]. Free radicals, like ROS, mediate damages to various membranes, including those enclosing the cell and the mitochondria. This can cause damages to cellular components, including lipids, fatty acids, proteins, and nucleic acids, finally leading to cell death and the loss of function of the electronic transport chain. The cytotoxic effect of AgNPs on treated THP-1 and AMJ-13 may be related to cellular damage that is mediated by oxidative stress. Similarly, the time-dependent manner of the increase in LDH release caused by AgNPs could be related to the disruption in the cell membrane, causing cellular enzymes, e.g., lactate dehydrogenase, to leak out [45]. 

### 3.3. Anti-Proliferative Activity of AgNPs

The cytotoxic effect of the prepared AgNPs against THP-1, HBL, and AMJ-13 cells was studied. The antitumor activity of the prepared AgNPs was tested by studying their capability of inhibiting the proliferation of malignant cells. The results showed highly significant toxic impacts of the prepared AgNPs toward THP-1 and AMJ-13 human cells. While, the results also showed a cytotoxic effect of AgNPs against the normal cell line (HBL cells). Evidence of the antitumor activity of AgNPs was obtained by studying their ability to block and inhibit the proliferation of tumor cells (Figure 3B), which showed that the AgNPs were capable of inhibiting the proliferation of cancer cell lines. The THP-1 and AMJ-13 cells were similarly affected by the exposure to AgNPs. The results suggest the ability of AgNPs to suppress the growth of cancer cell lines. Meanwhile, treating cancer cells with AgNPs for 72 h resulted in a significant reduction in their ability to proliferate (Figure 3C). The reduction of colony formation of cancer cells after continuous exposure to AgNPs for 24 h at a concentration (IC_50_) of 17.34 µgmL^−1^ should demonstrate that they have been killed (Figure 3D), suggesting that the AgNPs were picked up by cells, prompting them to undergo apoptosis. Therefore, the findings demonstrated that the AgNPs could induce cell death. Taken together, the results suggest a selective mode of inhibition by the AgNPs on THP-1 and AMJ-13 cells. 

### 3.4. AgNPs Induce Apoptosis in THP-1 and AMJ-13 Cells 

**Figure 4:**
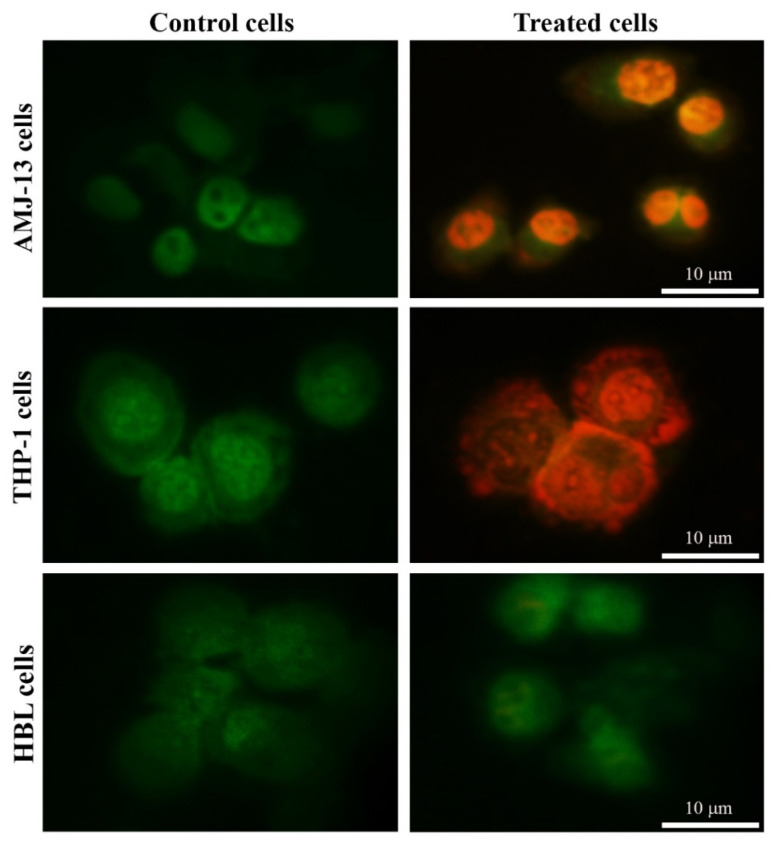
Apoptotic markers in AMJ-13, THP-1, and HBL cells treated with AgNPs at IC_50_ (17.34 µgmL^−1^) for 24 h and stained with AO/EtBr for 1 min. Untreated cells are shown with a normal structure. However, after treatment with AgNPs, apoptotic features are observed; the red color indicates apoptosis. Scale bar 10 µm.

**Figure 5:**
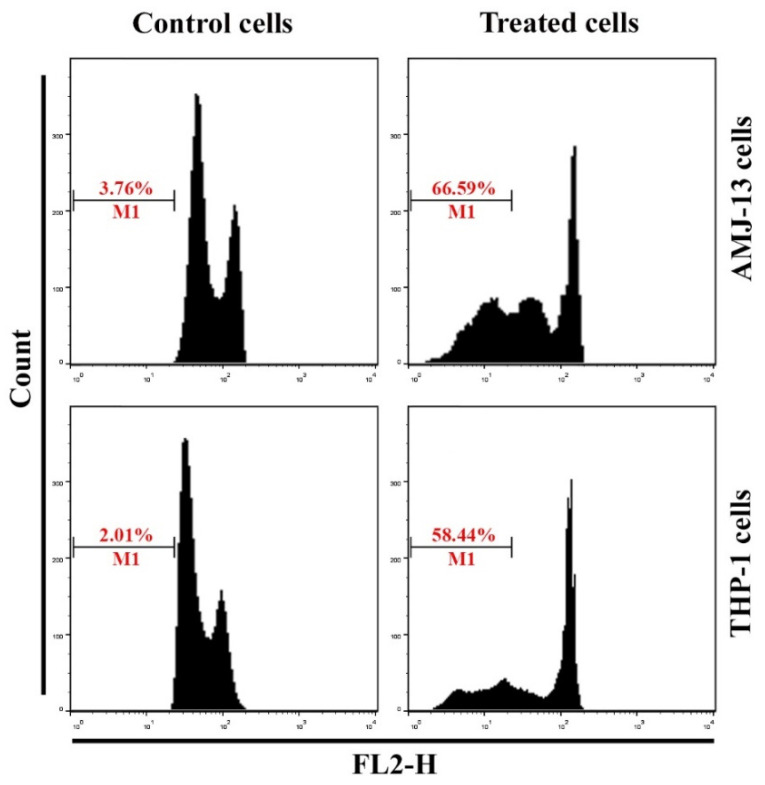
AgNPs induce apoptosis in AMJ-13 and THP-1 cells. Cells were exposed to AgNPs at IC_50_ (17.34 µgmL^−1^) for 24 h then stained with propidium iodide (PI). Sub-G_1_ phase of cells, flow cytometry data of AMJ-13 and THP-1 cells.

Apoptosis is a key mechanism for homeostasis, which controls the proportions of different cell types [46]. We hypothesized that the prepared AgNPs induce the apoptosis of treated THP-1, AMJ-13, and HBL cell lines, in a manner correlated with anti-proliferative activity. We studied whether the AgNPs exerted anti-proliferative effects toward leukemia (THP-1), breast cancer (AMJ-13), and normal (HBL) cell lines derived from humans, in a manner related to the stimulation of apoptosis. These cells were subjected to treatment with 17.34 µgmL^−1^ of AgNPs. The dual stain Ao/EtBr is a fluorescent mixed stain for detecting morphological changes in the nucleus, which produces different fluorescent colors. After treatment of THP-1 and AMJ-13 cells with AgNPs at IC_50_ concentrations for 24 h, dual cell staining and visualization under a fluorescence microscope were performed to determine the changes in nuclear morphology, as shown in Figure 4. Hence, cancer cells were treated with AgNPs, they demonstrated an increase in the disruption of the membrane and the vacuoles of the lysosomes, in comparison with that of untreated cells. While, no changes were shown in the normal cell line. The results revealed the strong cell death-causing capability of the prepared AgNPs, which is attributed to their high membrane penetrating potential. Aiming at deeper examination for the extent of cancer cell killing-induction capability of AgNPs, a mixture of acridine orange and ethidium bromide dyes was employed. The results demonstrated an intact nuclear structure, with a stable bright green color. Cancer cells subjected to AgNPs treatment exhibited membranes with lower integrity than those of the control cells. Apoptotic cells are typically known to demonstrate nuclei with red to orange color. In current study, we performed a further experiment to investigate the possible inductive role of AgNPs in apoptosis. We tested the DNA content at the sub-G_1_ phase by utilizing flow cytometry, following the staining of the cellular DNA of treated THP-1 and AMJ-13 cells with PI. The results demonstrated that the proportion of AgNP-treated cancer cells in the sub-G_1_ phase was increased from 3.67% to 66.59% in AMJ-13 cells and from 2.01% to 58.44% in THP-1 cells (Figure 5). Overall, the outcomes of this study demonstrated that the prepared AgNPs exerted an anti-proliferative impact via the stimulation of apoptotic cell death.

### 3.5. Impacts of AgNPs on Mitochondrial Membrane Potential 

**Figure 6:**
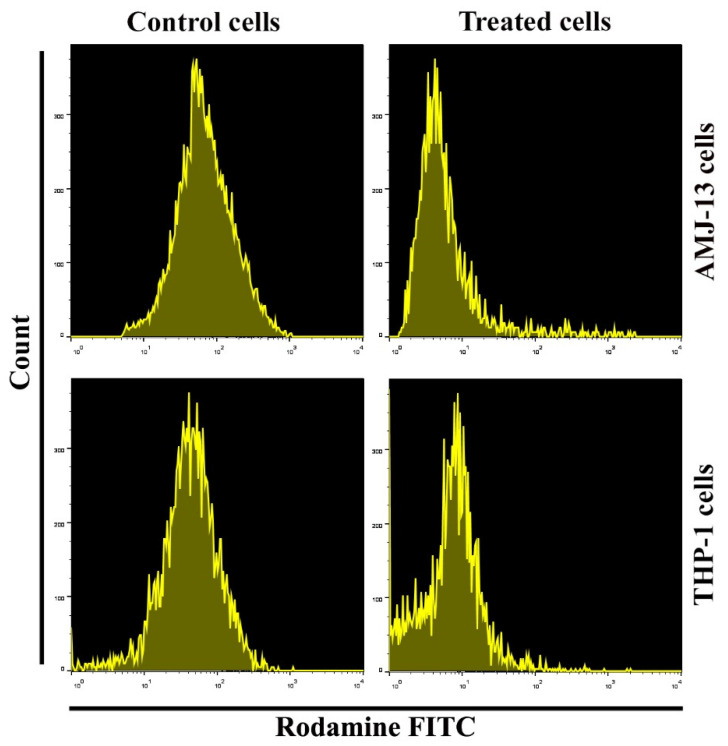
Dysfunction of MMP in AgNPs-treated AMJ-13 and THP-1 cells. Cells were exposed to AgNPs at IC_50_ (17.34 µgmL^−1^) for 24 h then stained with Rh123 dyes (10 M for 1 h at 37 °C). Flow cytometry data in cells treated with AgNPs. Rhodamine staining was employed to examine the effects on mitochondrial membrane potential.

Mitochondria play important functions in the induction of apoptotic mechanisms through various t stimulation signals of cell death. Representative changes include the depletion of mitochondrial membrane potential (∆ψm), with cytochrome c protein being released into the cytoplasm and causing the upregulation of caspase-3 via caspase-9 pathway. The level of mitochondrial membrane potential was examined by utilizing flow cytometry following the staining of cells with the Rh123 probe. The proportion of cancer cells undergoing apoptosis was determined after treatment with the prepared AgNPs. There was a remarkable increase of apoptosis due to AgNP treatment of THP-1 and AMJ-13 cells, as shown in Figure 6. These treated cells showed a significant decrease in Rh123 staining, which was confirmed to reflect the loss of the mitochondrial membrane potential, in comparison with compared with the case for the untreated cells. 

### 3.6. Potentials of AgNPs on Upregulation of p53 Expression 

**Figure 7:**
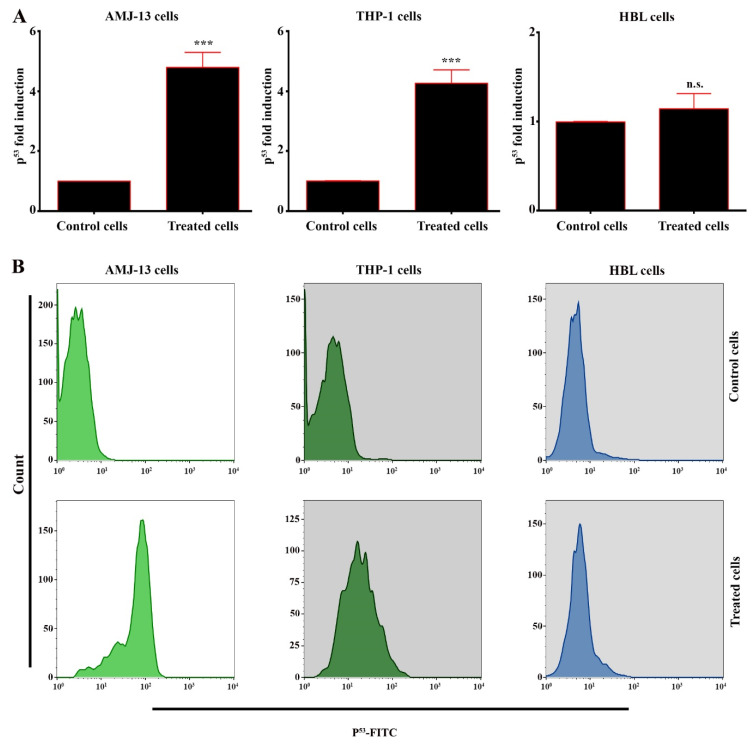
AgNPs upregulate p53 expression in AMJ-13 and THP-1 cells. (**A**) Cells were left untreated or treated with AgNPs at IC_50_ (17.34 µgmL^−1^) for 24 h. Asterisks indicate statistically significant difference from untreated cells. (**B**) Represented Flow cytometry data in cells treated with AgNPs as in (**A**). Results are represented as mean ± SD.*** *p* < 0.001, ns: Non-significant.

Given that the above results indicated that the prepared AgNPs have the capability of inducing a mitochondria-mediated apoptotic impact, the expression of p53 was tested by real-time PCR and flow cytometry assays. Treatment of the THP-1 and AMJ-13 cells with AgNPs at the IC_50_ concentration for 24 h caused a significant increment in p53 expression level in comparison with that of the untreated cells, as shown in Figure 7A. p53 activity was investigated by flow cytometry. The results illustrated in Figure 7B reveal that the cells treated with AgNPs at a concentration 17.34 µgmL^−1^ have a high shift toward the right for P53 designated. We also detected the levels of P53, where the treatment of THP-1 and AMJ-13 cells with the AgNPs led to significantly increased protein expression levels of p53 compared with those of the control cells. p53 signaling was markedly increased, which demonstrated that the AgNPs can induce apoptosis via the p53 pathway. 

### 3.7. AgNPs increase Autophagy Following LPS+ATP Treatment 

**Figure 8:**
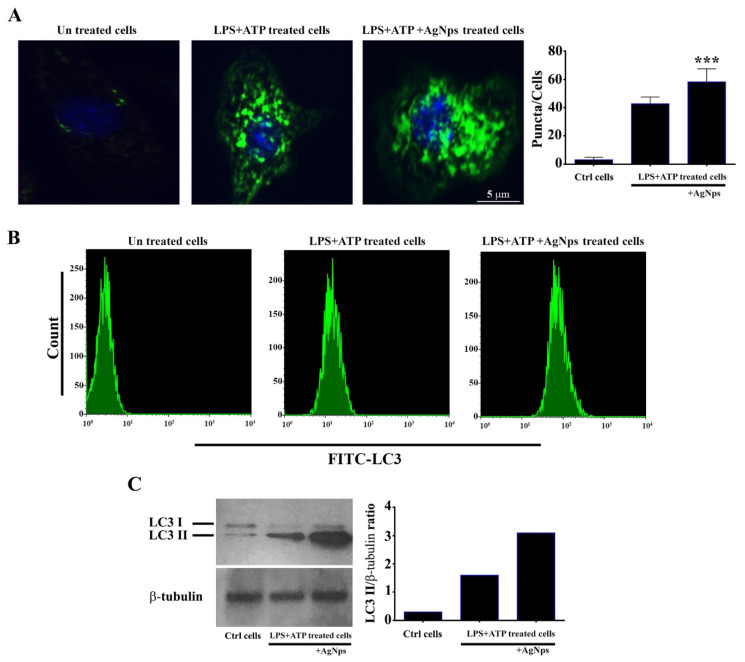
AgNPs augmented autophagy following LPS+ATP treatment of cells. (**A**) Immunofluorescence graphs of LC3 in bone marrow-derived macrophages (BMDMs). Cells were treated, or not, with LPS+ATP in the presence and absence of 5 µgmL^−1^ AgNPs. Fixation, permeabilization, and staining with DAPI were performed to visualize nuclei (blue); autophagosome staining appears in green. Scale bar, 5 µm. Graph represents numbers of LC3 puncta in cells after treatment with LPS+ATP, with and without AgNPs treatment (5 µgmL^−1^); quantification of the results was performed with Image J software. Asterisks refer to statistical difference between cells with and without treatment with 5 µgmL^−1^ AgNPs. Results are expressed in mean ± SD. *** *p* < 0.001. (**B**) Flow cytometry analysis for LC3 protein in BMDMs following treatment with LPS+ATP, with and without treatment with 5 µgmL^−1^ AgNPs. (**C**) Western blot analysis of LC3-I and LC3-II proteins. Cells left untreated as control or treated with LPS+ATP in absence and absence of AgNPs at a concentration of 5 µgmL^−1^. Graph represented densitometry quantification of the LC3-II/b-tubulin ratio, as indicated.

**Figure 9:**
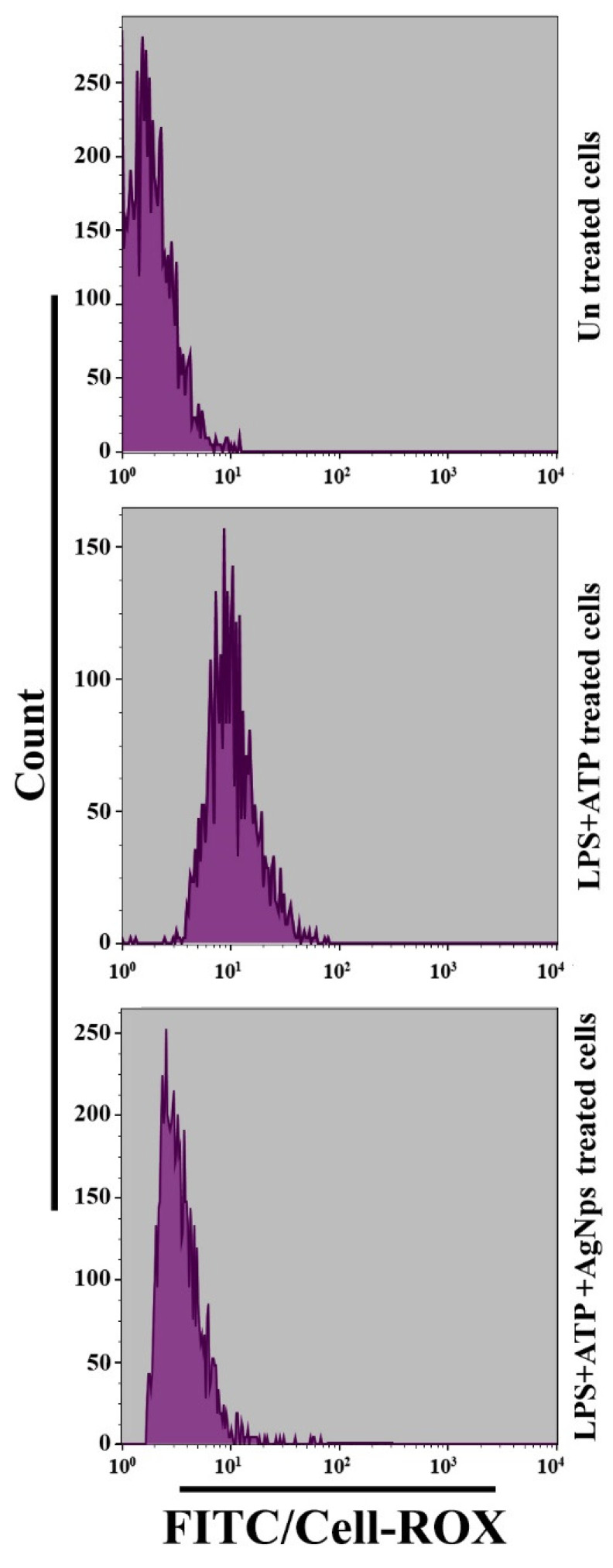
AgNPs reduce activation of ROS in cells. Cells were treated, or not, with LPS+ATP with and without treatment with 5 µgmL^−1^ AgNPs. The ROS accumulation was determined by flow cytometry.

We hypothesized that the prepared AgNPs would augment autophagy following LPS+ATP treatment of BMDM cells. BMDMs were treated with LPS at a concentration of 500 µgmL^−1^ for 24 h followed by treatment with ATP at a concentration of 5 mMmL^−1^ for 30 min, with or without treatment with AgNPs (5 µgmL^−1^). The ability of the prepared AgNPs to increase autophagy was measured using immunofluorescence, as shown in Figure 8A, and flow cytometry, as shown in Figure 8B. The induction of autophagy in the cells subjected to treatment with LPS+ATP in the presence of AgNPs was investigated by measuring the production of the lapidated form of LC3 (LC3 II) by Western blot assay, as demonstrated in Figure 8C. The results showed that the prepared AgNPs increased autophagosomes formation as well as LC3II protein level. Autophagy is known to be able to reduce the formation of ROS, which exert important functions related to their mediation of the intrinsic apoptotic pathway. Therefore, we studied the possible mechanism by which AgNPs can induce autophagy. We hypothesized that the AgNps augment autophagy through the reduction of ROS production. To test our hypothesis, we measured ROS level in cells following treatment with LPS+ATP, with or without the addition of AgNPs. Flow cytometry was employed to investigate ROS level production in cells pre-treated with AgNps for 1 h and then treated with LPS+ATP. The results showed that LPS+ATP significantly increased, while AgNPs reduced, ROS production (Figure 9). Taken together, these results demonstrated that autophagy induced by LPS+ATP decreases ROS production. 

### 3.8. AgNPs Inhibit IL-1β, Caspase-1, ASC, and NLRP3 Inflammasome Activation

**Figure 10:**
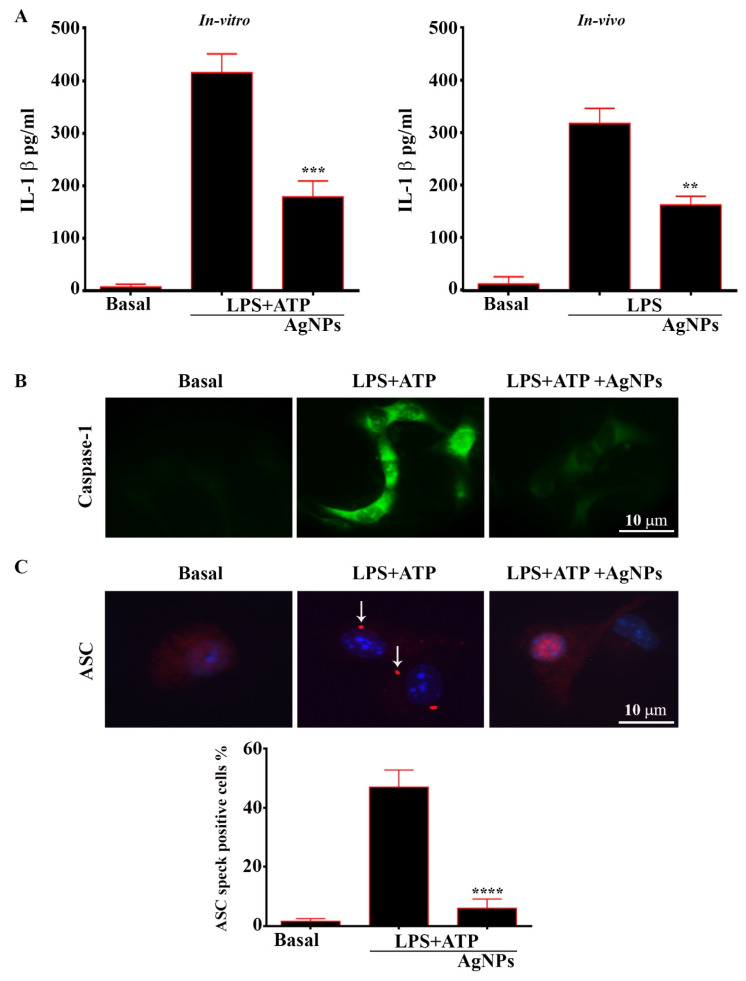
AgNPs inhibit inflammasome activation. (**A**), AgNPs inhibit IL-1-beta secretion in-vitro and serum of treated mice (in-vivo). Asterisks indicate statistically significant difference from LPS+ATP-treated cells in the presence and absence of AgNPs at a concentration of 5 µgmL^−1^. Results are presented as mean ± SD. ** *p* < 0. 01, *** *p* < 0.001, **** *p* < 0.0001. (**B**), Immunofluorescence images of Caspase-1 in cells. Cells were left untreated or treated with LPS+ATP in the presence and absence of AgNPs at a concentration of 5 µgmL^−1^. Cells were fixed, permeabilized, and stained with DAPI to visualize nuclei (blue); Caspase-1 staining is shown in green. Scale bar, 10 µm. (**C**), Immunofluorescence images of ASC. Cells were treated with LPS+ATP in the presence and absence of AgNPs at a concentration of 5 µgmL^−1^. Cells were fixed, permeabilized, and stained with DAPI to visualize nuclei (blue); ASC staining is shown in red. The white arrow represents ASC specks Scale bar, 10 µm. Graph represented Percentage of cells containing ASC speck.

**Figure 11:**
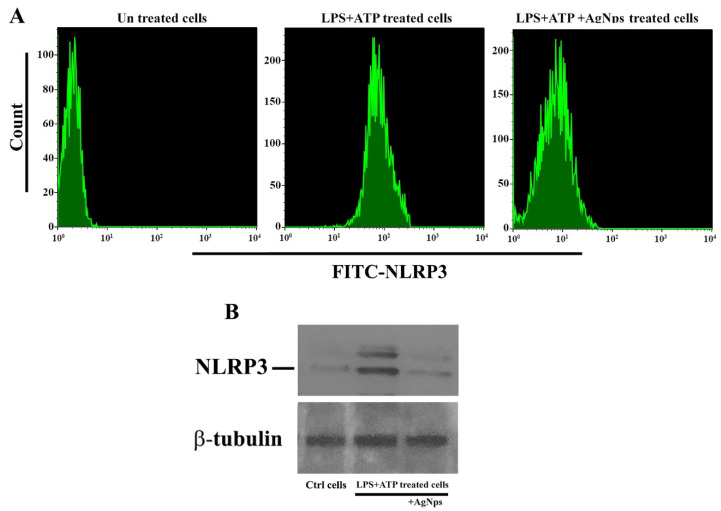
AgNPs reduce NLRP3 inflammasome activation. (**A**), Flow cytometry analysis for NLRP3 in BMDMs following treatment with LPS+ATP, with and without treatment with 5 µgmL^−1^ AgNPs. (**B**), Westren blot analysis of NLRP3 protein. Cells left untreated as control or treated with LPS+ATP in absence and absence of AgNPs at a concentration of 5 µgmL^−1^.

We hypothesized that the AgNPs’ influence on autophagy might be able to change inflammasome activation in both in vitro and in vivo models. The impact of the addition of AgNPs to BMDMs was investigated. Thereafter, the cells were subjected to treatment with 500 µgmL^−1^ LPS + 5 mM ATP and compared with untreated basal cells. The BMDM cells pretreated with AgNPs showed significant decreases in IL-1β levels, as compared to those treated with LPS and ATP. Meanwhile, the results of the in vivo model confirmed the ability of AgNPs to reduce IL-1β, as shown in Figure 10A. The activation of inflammasome is dependent on 2 main signals. Firstly, through the induction of NF-κ B which results in the production of pro-IL-1β and pro-caspase-1. Secondly, is required for the proper assembly of the inflammasome complex, which leads to the recruitment of pro-caspase-1. In the present work, we studied the impact of AgNPs on Caspase-1 activation and ASC oligomerization; cells were exposed to LPS + ATP, with and without treatment with AgNPs. The results showed the ability of AgNPs to inhibit Caspase-1 activity (Figure 10B). Significantly, pre-treatment of cells with AgNPs following LPS+ATP did not affect TNF-α production (data not shown), suggesting that AgNPs could reduce the release of IL-1β via their interference with signal-2. In addition, AgNPs inhibits Caspase-1 activity. In the present investigation, we tested the inhibitory influence of AgNPs on the adaptor protein ASC. To examine this opportunity, we tested NLRP3 inflammasome complexes as ASC specks using the immunofluorescence assay. Cells treated with LPS+ATP contained an increased number of ASC specks, as shown in Figure 10C. Nevertheless, AgNPs-treated cells demonstrated a significantly lower number of ASC specks. Taken together, the results demonstrated that AgNPs impede the activation of the inflammasomes via the inhibition of ASC oligomerization. Then, we studied the role of AgNPs in enhancing NLRP3 degradation via autophagy. We measured the level of NLRP3 inflammasome following LPS+ATP treatment of cells, with or without treatment with 5 µgmL^−1^ AgNPs. Our outcomes revealed a decrease in the level of NLRP3 inflammasome in BMDM cells subjected to combined treatment with LPS+ATP and AgNPs. We tested the NLRP3 by utilizing Flow cytometry (Figure 11A), and confirmed our results using Western blot assay, as demonstrated in Figure 11B. 

**Figure 12:**
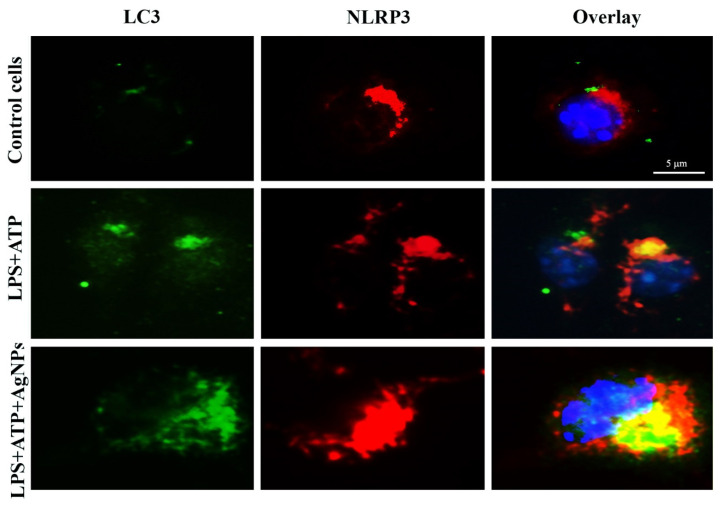
AgNPs increase the targeting of NLRP3 by autophagy. Confocal images of LC3 in BMDMs (green): Cells were left untreated as a control or treated with LPS+, with and without treatment with 5 µgmL^−1^ AgNPs. Staining of BMDMs was with anti-NLRP3 antibody (red) and staining of nuclei was with DAPI. Co-localization of the LC3 and NLRP3 staining reveals the merged LC3 and NLRP3 signals; co-localized regions appear in yellow. Scale bar, 5 mm.

To investigate how the NLRP3 is localized intra-cellularly and how its pattern of secretion is regulated by autophagy, LPS+ATP-treated BMDM cells were dyed using anti-NLRP3 and anti-LC3 antibodies. Visualization of the treated cells was conducted under confocal microscopy. BMDM cells treated with both LPS+ATP and AgNPs exhibited increases in autophagosome formation and intracellular NLRP3 in LC3 autophagosomes. The results showed the occurrence of substantial co-localization between LC3 and NLRP3 in the presence of AgNPs, as shown in Figure 12. These findings show that the AgNPs induced the augmentation of autophagy as well as the degradation of IL-1β and NLRP3 inflammasome. Thus, this work demonstrates that autophagy has a central role in controlling the release of IL-1β and NLRP3 within LPS+ATP-treated BMDM cells.

### 3.9. Inhibition of Autophagy Reduces the Role of AgNPs in Inflammasome Activation

**Figure 13:**
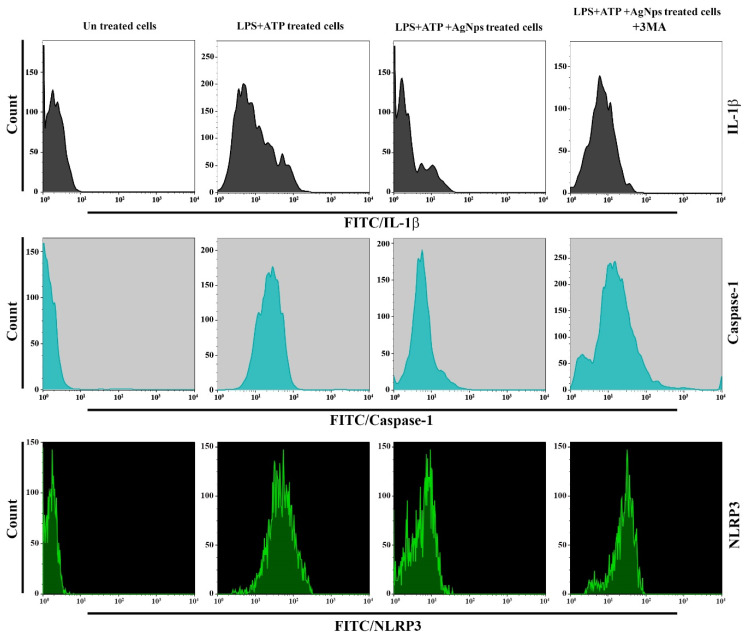
Inhibition of autophagy blocks the role of AgNPs in inflammasome activation. Cells were left untreated as a control or treated with LPS+ATP, with and without treatment with 5 µgmL^−1^ AgNPs, with 3-MA at concentration 10 mM. Flow cytometry analysis was performed for the indicated proteins.

In the current study, we also tested the impacts of the inhibition of autophagy on inflammasome activation in cells subjected to combined treatment with both LPS+ATP and AgNPs. Impedance of autophagy following the use of 3-methyladenine (3-MA) resulted in higher activation of inflammasomes in cells after combined treatment with both LPS+ATP and AgNPs. The findings demonstrated that the levels of IL-1 β, Caspase-1, and NLRP3 were markedly increased, as shown in Figure 13. These findings demonstrate that the absence of autophagy leads to higher activation of inflammasomes, following LPS+ATP treatment in the presence of AgNPs. 

## 4. Conclusions

In this study, AgNPs were prepared using a green synthesis method. Then, we investigated their role as an anticancer agent against THP-1, AMJ-13, and HBL cells, in terms of inhibiting cell proliferation and inducing apoptosis. We found that the AgNPs exhibited potent anti-proliferative and pro-apoptotic impacts on the THP-1 and AMJ-13 cell lines, which could involve both the death receptor and the mitochondrial pathways. Silver nanoparticles reduced Caspase-1 and ASC and decreased IL-1beta levels in both in vivo and in vitro models. Meanwhile, the administration of AgNPs significantly decreased NLRP3 inflammasome activation. The results demonstrate that the AgNPs increased the process of autophagy, leading to the inhibition of NLRP3 inflammasome activation and IL-1b release. The increase of autophagy in the presence of AgNPs caused IL-1beta and NLRP3 to be targeted by autophagy autophagosomes. This work described a novel pathway by which AgNPs increase autophagy, controlled by the inflammasome pathway via two different mechanisms. The first one is by regulating the activation of IL-1 β, caspase-1, and ASC, while the second is by targeting NLRP3 for degradation. The results confirmed that AgNPs could be used for treating different types of cancers and promoting immune system functions.
